# Development of a sample preparation procedure for Sr isotope analysis of Portland cements

**DOI:** 10.1007/s00216-021-03821-7

**Published:** 2022-01-14

**Authors:** Anera Kazlagić, Francesco F. Russo, Jochen Vogl, Patrick Sturm, Dietmar Stephan, Gregor J. G. Gluth

**Affiliations:** 1 Division 1.1 Inorganic Trace Analysis, Federal Institute for Materials Research and Testing, Richard-Willstäter-Straße 11, 12489 Berlin, Germany; 2 Division 7.4 Technology of Construction Materials, Federal Institute for Materials Research and Testing, Unter den Eichen 87, 12205 Berlin, Germany; 3 Department of Civil Engineering, Building Materials and Construction Chemistry, Technische Universität Berlin, Gustav-Meyer-Allee 25, 13355 Berlin, Germany

**Keywords:** Cement, Clinker, Sample preparation, Strontium isotopes, Provenancing

## Abstract

The ^87^Sr/^86^Sr isotope ratio can, in principle, be used for provenancing of cement. However, while commercial cements consist of multiple components, no detailed investigation into their individual ^87^Sr/^86^Sr isotope ratios or their influence on the integral ^87^Sr/^86^Sr isotope ratio of the resulting cement was conducted previously. Therefore, the present study aimed at determining and comparing the conventional ^87^Sr/^86^Sr isotope ratios of a diverse set of Portland cements and their corresponding Portland clinkers, the major component of these cements. Two approaches to remove the additives from the cements, i.e. to measure the conventional ^87^Sr/^86^Sr isotopic fingerprint of the clinker only, were tested, namely, treatment with a potassium hydroxide/sucrose solution and sieving on a 11-µm sieve. Dissolution in concentrated hydrochloric acid/nitric acid and in diluted nitric acid was employed to determine the ^87^Sr/^86^Sr isotope ratios of the cements and the individual clinkers. The aim was to find the most appropriate sample preparation procedure for cement provenancing, and the selection was realised by comparing the ^87^Sr/^86^Sr isotope ratios of differently treated cements with those of the corresponding clinkers. None of the methods to separate the clinkers from the cements proved to be satisfactory. However, it was found that the ^87^Sr/^86^Sr isotope ratios of clinker and cement generally corresponded, meaning that the latter can be used as a proxy for the clinker ^87^Sr/^86^Sr isotope ratio. Finally, the concentrated hydrochloric acid/nitric acid dissolution method was found to be the most suitable sample preparation method for the cements; it is thus recommended for ^87^Sr/^86^Sr isotope analyses for cement provenancing.

**Graphical abstract:**

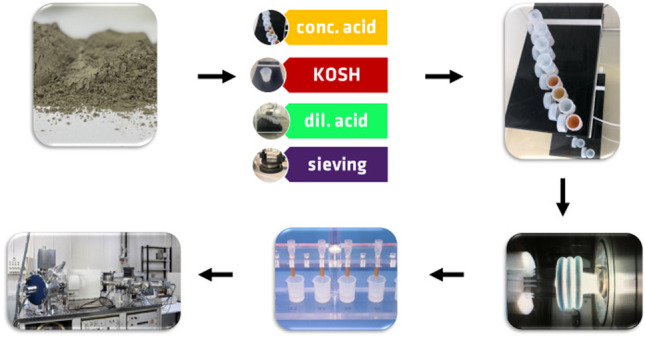

**Supplementary Information:**

The online version contains supplementary material available at 10.1007/s00216-021-03821-7.

## Introduction

Concrete is the most important artificial material in the world in terms of produced mass, and it is considered to be the basis for our built environment [1]. Since the properties of concretes are dominated by their key compound, cement [2], devising a way to determine its origin, known as provenancing, is of great importance. Provenance studies of concrete and cement are required for failure research, damage assessment, and resulting liability issues related to concrete structures, and they are also important in forensic science [3–8].

To determine the origin of cement, it is important to understand its composition and production process. At present, ordinary Portland cement (OPC) is the most widely used cement type, suitable for most purposes of concrete construction. As specified in European Standard EN 197–1, Portland cement (CEM I) contains at least 95 wt.% of Portland clinker (disregarding the calcium sulfate addition). The clinker is a complex mixture of calcium silicates, calcium aluminates, and several minor phases produced from limestone, chalk, marl, clays, shale, and partly other minor components in a rotary kiln [2, 9]. The remaining 5 wt.% or less of the CEM I are minor additional constituents, as specified in EN 197–1; often, limestone is used. To control the setting of the cement after addition of water, calcium sulfate is added to the cement in small quantities. Usually, natural gypsum and/or anhydrite, industrially processed chemical gypsum, or flue gas desulfurisation (FDG) gypsum is employed [10]. Each of these materials contains strontium, an element whose isotopes provide information on the geographical origin, and, therefore, is highly valuable for provenance studies.

The potential of conventional ^87^Sr/^86^Sr isotope ratios, hereafter referred to as ^87^Sr/^86^Sr isotope ratios, for unravelling the origin of cement was first mentioned by Graham and co-authors [11]. The advantage of using radiogenic isotope systems such as the Sr isotope system is that the results can be linked to the geographic origin of raw materials with more confidence than the results of other approaches such as elemental fingerprinting. This is partly due to the fact that the elemental composition of a cement may be considerably influenced by changes in the environment as well as changes of the production process [3]. As ordinary Portland cement consists mainly of Portland clinker, ^87^Sr/^86^Sr isotope ratios of cement and clinker might be expected to be very similar. However, since the additional cement constituents, as well as the added calcium sulfates, can come from very different geological backgrounds or industrial processes, their effect on the ^87^Sr/^86^Sr isotope ratio of the resulting cement may be considerable. The magnitude and significance of this effect have not been explored previously. Therefore, the present study compared the ^87^Sr/^86^Sr isotope ratios of a number of cements and clinkers of varying geographical origins and tested approaches to separate the clinker from its parent cement to enable measuring its ^87^Sr/^86^Sr isotope ratio for provenancing of the cement.

## Materials and methods

### Cement and clinker samples

Fifteen cements (all ordinary Portland cement, CEM I according to DIN EN 197–1) and the fifteen corresponding Portland clinkers used to produce these cements were obtained from the respective cement producers. All cements contained the four major clinker phases alite (Ca_3_SiO_5_), belite (Ca_2_SiO_4_), aluminate (Ca_3_Al_2_O_6_), and ferrite (Ca_2_AlFeO_5_) in comparable amounts, as determined by X-ray diffraction (XRD) analysis (see “X-ray diffraction measurements of non-treated and sieved samples” for experimental conditions). However, the amount(s) of the calcium sulfates, i.e. gypsum (CaSO_4_·2H_2_O), hemihydrate (CaSO_4_·0.5H_2_O), and/or anhydrite (CaSO_4_), differed considerably between the cements, as will be discussed in “XRD results”. Nine cements and clinkers originate from Germany, two from Serbia, one from Greece, one from Bosnia and Herzegovina, one from Kosovo, and one from North Macedonia. The rationale for the investigation of cements and clinkers from different countries was to include materials with a wide range of isotope ratios of the raw materials as well as different CEM I production plants. The obtained sample units (usually ~ 0.5–1 kg) were divided into smaller portions using standard procedures. The samples were stored in PP beakers. The sample mass for ^87^Sr/^86^Sr isotope ratio analysis was ≈ 100 mg.

### Chemicals

HNO_3_ (65–68% *v*/*v*) and HCl (≈ 30% *v*/*v*) were purchased as *pro analysis* grade acids (Chemsolute®, Th. Geyer, Berlin, DE) and were further purified by double sub-boiling distillation. For the potassium hydroxide/sucrose treatment (“KOSH treatment”), sucrose (SERVA Electrophoresis GmbH, Heidelberg, DE, analytical grade, min. 99 wt.% purity), KOH (Merck KGaA, Darmstadt, DE, *pro analysi*), and Milli-Q water (Milli-Q Advantage A10 System, Merck KGaA, Darmstadt, DE) were used. For Sr purification and matrix separation, Sr·Spec™ resin (100–150 µm, Eichrom Technologies Inc, Lisle, IL, USA) was employed.

### Sample preparation methods: overview

All procedures such as weighing, sample dissolution, and analyte separation were performed in a metal-free clean laboratory with ISO class 6 at the Federal Institute for Materials Research and Testing (BAM). Crushing and grinding of the samples were performed in a normal laboratory environment.

Clinker samples were crushed and ground before digestion in a 1:1 *v/v* mixture of concentrated HCl and HNO_3_ on the hotplate (130 °C) for 48 h.

The cement samples were ground as well (except for those being applied to the sieving procedure) and afterwards prepared using four different procedures described below (“Concentrated hydrochloric acid/nitric acid dissolution” to “KOSH treatment”). Subsequently, the solutions containing the samples were evaporated until dryness, the precipitate was redissolved in 2% *w*/*w* HNO_3_, and afterwards, an aliquot was analysed by inductively coupled plasma mass spectrometry (ICP-MS) to determine the Sr mass fraction. Then, a subsample containing approximately 2 µg Sr was used for matrix separation by column chromatography using the Sr resin. The isolated Sr fraction was then dried, redissolved, and loaded on Re filaments, which were used for multi collector thermal ionisation mass spectrometry (MC-TIMS) measurements.

**Fig. 1:**
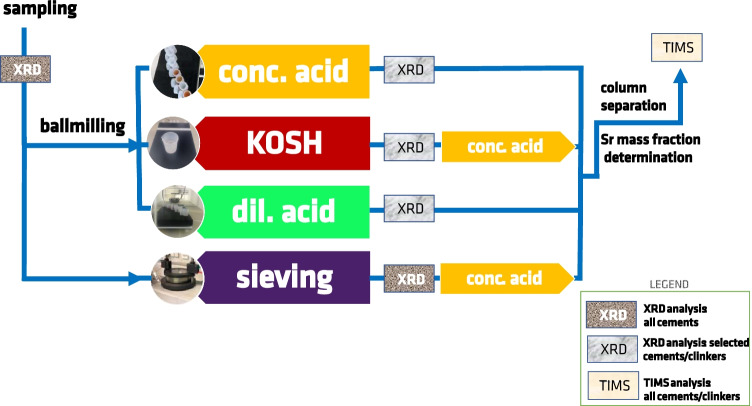
Schematic representation of the four sample preparation procedures for cement samples. *Conc. acid* refers to dissolution in concentrated hydrochloric acid/nitric acid, *KOSH* refers to selective dissolution of clinker phases, *dil. acid* refers to dissolution in dilute nitric acid, and *sieving* is dry sieving performed on a 11-µm sieve. *XRD* refers to X-ray diffraction and *TIMS* refers to thermal ionisation mass spectrometry

To evaluate the sample preparation procedures, the precipitates after each digestion treatment were analysed by XRD and phase identification. In addition, to check the outcomes of the sieving treatment (“Sieving”), the sieved and non-treated cement samples were analysed by XRD as well. Figure 1 summarises the experimental programme.

### Concentrated hydrochloric acid/nitric acid dissolution

The concentrated hydrochloric and nitric acids were chosen because of their strong acidic character and consequent ability to dissolve cement phases. After ball milling, each CEM I sample was weighed (~ 100 mg) in Savillex® beakers. The next step was the addition of conc. HNO_3_ and conc. HCl (2 mL each). The beakers were closed, agitated by hand, and sonicated (20 min) and digestion was performed on a hotplate (130 °C) for 48 h. The sample preparation using concentrated HNO_3_ and HCl 1:1 *v/v* is abbreviated as “conc. acid”.

### Dilute nitric acid dissolution

The dissolution with 1 mol·L^−1^ HNO_3_ was already used by Graham and co-authors in their cement provenancing study [11], where it has proven effective for the identification of cement from hardened concrete samples. This approach was also used by Kasamatsu and co-authors [7] for analysing nitric acid-soluble components in the fragments of concrete for forensic issues.

In the present study, the cement samples were first ground in a planetary ball mill, weighed, and suspended in 4 mL of 1 mol·L^−1^ HNO_3_. The beakers were closed, agitated by hand, sonicated (20 min), and heated on a hotplate (130 °C) for 48 h. In the text below, the treatment by 1 mol·L^−1^ HNO_3_ is abbreviated as “dil. acid”.

### Sieving

The rationale for the application of sieving was that the clinker particles and the calcium sulfates in cements have different particle size distributions due to their different grindability. Portland clinker emerges from the kiln as rounded granules or irregularly shaped lumps, in either case with a dimension of about 3–30 mm. These are subsequently blended and ground with the calcium sulfates. Due to different hardnesses, the different cement components are enriched in fractions with different particle sizes. The harder the compound, the more is found in the larger particle size fraction; gypsum and its dehydration products are concentrated in the finer fractions [2]. Theoretically, this property should allow to separate the sulfates from the clinker by sieving and subsequently collecting the fraction with particles greater than a specified size.

The samples were sieved on a 11-µm sieve (Atechnik GmbH, Leinburg, DE) using a sieving machine (AS 200 control, Retsch GmbH, Haan, DE) and a setting of 2.52-mm amplitude for 20 min. Ideally, sieving should separate the coarse particles from the fine particles, with the coarser fraction remaining on the sieve being mainly composed of clinker. The so-obtained coarser fraction, of course, needs to be digested for further Sr isotope analysis. Therefore, these coarser fractions were dissolved in conc. acid as described in “Concentrated hydrochloric acid/nitric acid dissolution”. The whole applied procedure is abbreviated as “sieving”, except for the XRD results, where “sieving” refers to samples analysed immediately after sieving.

### KOSH treatment

A treatment with a potassium hydroxide/sucrose solution (the so-called KOSH solution) to selectively dissolve aluminate and ferrite phases from Portland cement, leaving alite and belite phases undissolved, was described by Gutteridge [12]. In the present context, the KOSH treatment was applied to remove the additives from the cements, while the concomitant dissolution of aluminate and ferrite was unavoidable.

Before the KOSH treatment, the cements were ground in a planetary ball mill (Pulverisette 5, Fritsch) to particle sizes ≤ 65 µm. The KOSH solution was prepared by weighing and mixing potassium hydroxide, sucrose, and water, using the procedure described by Gutteridge [12], except for slight modifications concerning smaller sample quantities while maintaining the same weight proportions as in the original procedure. Instead of filtration, we used centrifugation as the separation technique. The ball-milled CEM I samples (≈ 1 g) were mixed with KOSH solution (previously prepared by mixing 37.5 mL of Milli-Q H_2_O with 3.75 g of KOH and 3.75 g of sucrose) at 95 °C and stirred until the solution turned pale yellow. The solution was then centrifuged; the residue was washed two times with water and one time with methanol, with centrifuging steps in between. The supernatant was removed by pipetting, and the subnatant was dried in the oven overnight (40 °C). For the subsequent determination of their ^87^Sr/^86^Sr isotope ratios, the dry residues were then dissolved in conc. acid as described in “Concentrated hydrochloric acid/nitric acid dissolution”. The whole procedure is abbreviated as “KOSH”, except for the XRD results, where “KOSH” refers to samples analysed after KOSH treatment before digestion in conc. acid.

## Analytical techniques

### X-ray diffraction measurements of non-treated and sieved samples

All samples of the non-treated and the sieved cements were prepared for XRD measurements by filling the sample powder into the cylindrical cavity of a standard polyvinylchloride sample holder and gently compressing it by using a glass plate until the sample surface became aligned with the sample holder surface.

The XRD measurements were performed on a D8 ADVANCE diffractometer (Bruker AXS, DE) in Bragg–Brentano geometry under the following conditions: Cu Kα radiation (*λ* = 1.54187 Å); X-ray tube: 40 kV and 40 mA; step size: 0.02° 2*θ*; scanning rate: 2.4° 2*θ* min^−1^; LYNXEYE XE-T detector.

### X-ray diffraction measurements of the residues after treatment

Five of fifteen cement and clinker samples (namely 3022, 3028, 3050, 3063, 3078) were chosen to analyse their residues by XRD after different preparation methods. The selection was based on a comparison of the phase assemblages of all cements. The chosen samples contained different amounts of calcium sulfates, i.e. the contents of gypsum and anhydrite were either zero, close to the maximum of all cements, or intermediate. Therefore, the XRD results of the five cements are representative for the processes occurring during treatments in all fifteen cement samples.

The KOSH, conc. acid, and dil. acid residues were ground manually with mortar and pestle (agate) before the XRD measurements. Since the available sample masses were low, the resulting powders of the KOSH residues were dusted on a flat (no cavity), single-crystal Si sample holder, cut to give no reflections in the measurement range. The dil. acid residues and the conc. acid residues had a jelly-like consistency and were smeared on the centre region of the Si sample holder. These latter samples had a hygroscopic character, and as a result, the samples turned partially or completely into viscous liquids during the XRD measurements.

The XRD patterns were recorded on an Ultima IV diffractometer (Rigaku, Japan) in Bragg–Brentano geometry under the following conditions: Cu Kα radiation (*λ* = 1.54187 Å); X-ray tube: 40 kV and 40 mA; step size: 0.01° 2*θ*; scanning rate: 0.2° 2*θ* min^−1^; scanning range: 5–65° 2*θ*; divergence slit 1/2° (in plane), 10 mm (axial); strip detector D/teX Ultra.

### Conventional ^87^Sr/^86^Sr isotope analysis (MC-TIMS analysis)

Approximately 100 mg of each cement and clinker sample was weighted in a Savillex® beaker and prepared according to the previously described methods. After determination of the Sr mass fraction (by iCAP-Q ICP-MS, Thermo Fisher Scientific, Bremen, DE), an aliquot of the sample was transferred to a new Savillex® beaker and dried on a hotplate. After drying, the sample was redissolved in 3 mol·L^−1^ HNO_3_ (1 mL). An aliquot of this solution containing 2 μg strontium was taken to perform a strontium matrix separation using the water suspension of Sr·Spec™ resin (350 µL) in polyvinylchloride columns (6 mm inner diameter, 4 cm long). The resulting strontium fraction was evaporated to dryness and redissolved in nitric acid such that a final strontium mass fraction of 100 ng·μL^−1^ was obtained, which could be directly used for loading 1 µL of the sample on Re filaments, together with TaF_5_ activator for enhancing the ionisation. Strontium isotope analyses were carried out by MC-TIMS at BAM in Berlin using a Sector 54 instrument (Micromass Ltd., Manchester, UK), in a dynamic multi-collection mode via an automatic measurement procedure. The raw measured data were corrected for interfering Rb and mass fractionation (^86^Sr/^88^Sr = 0.1194) and finally were normalised to a NIST SRM 987 ^87^Sr/^86^Sr ratio of 0.71025 [13], which is also the median of more than thousand published results listed in the GeoRem database [14]. Furthermore, NASS-6 seawater reference material was used as a control sample.

## Results and discussion

### XRD results

#### XRD results of non-treated and sieved cements

The diffractograms of the cements before and after sieving (see Supplementary Information (ESM), Figs. S1–S3) reveal that the cements exhibited similar phase assemblages as regards the major clinker phases alite (Powder Diffraction File [15] [PDF] # 01–073-0599), belite (PDF # 01–086-0398), aluminate (PDF # 00–038-1429), and ferrite (PDF # 00–030-0226). In addition, in some of the cements, minor amounts of quartz (SiO_2_; PDF # 00–046-1045) and calcite (CaCO_3_; PDF # 01–086-0174) impurities were identified.

More significant differences existed regarding the calcium sulfates in the cements, namely anhydrite (PDF # 01–072-0916), hemihydrate (PDF # 01–083-0438), and gypsum (PDF # 00–033-0311). Anhydrite exhibited the strongest intensities, i.e. anhydrite was the main sulfate in most cases (cements 3022–3029, 3032). The pertinent cements additionally contained minor amounts of hemihydrate, noticeable as a shoulder at 14.74° 2*θ* in the diffractograms. Gypsum was the main sulfate in cement 3063, which was the only sample with no anhydrite signals. In all other samples, gypsum occurred either as a minor component or this phase was not present at all (cements no. 3022, 3029, 3030, 3078). The differences between the cements result from different additions of sulfates during cement production as well as partial dehydration of the hydrated calcium sulfates during cement milling. Their amounts are usually optimised and adapted to the clinker composition by the cement producer to achieve optimum setting and other properties of the final material [2].

Comparison of the diffractograms of the non-treated (un-sieved) and the sieved cements (see ESM, Figs. S1–S3) shows that in no case sieving removed the calcium sulfates from the cements. Instead, the sulfate contents of the cements (as indicated by the heights of the reflections of the sulfates, relative to the intensity of the reflection of alite at 32.20° 2*θ*, i.e. *d* = 2.78 Å) were comparable before and after sieving, and in some cases, the fraction of anhydrite or gypsum was even higher after sieving (e.g. cements 3022 and 3063). The present results clearly show that the applied sieving procedure did not separate additives from clinker in the cement. The underlying reasons could not be conclusively clarified. However, a likely explanation is that the fine calcium sulfate particles stuck to the clinker particles during sieving due to physical bonding. Therefore, separation based on a size differentiation with the proposed method is not suitable to separate coarser clinker particles from the finer particles, such as added calcium sulfates.

#### XRD results of the residues after KOSH treatment

**Fig. 2:**
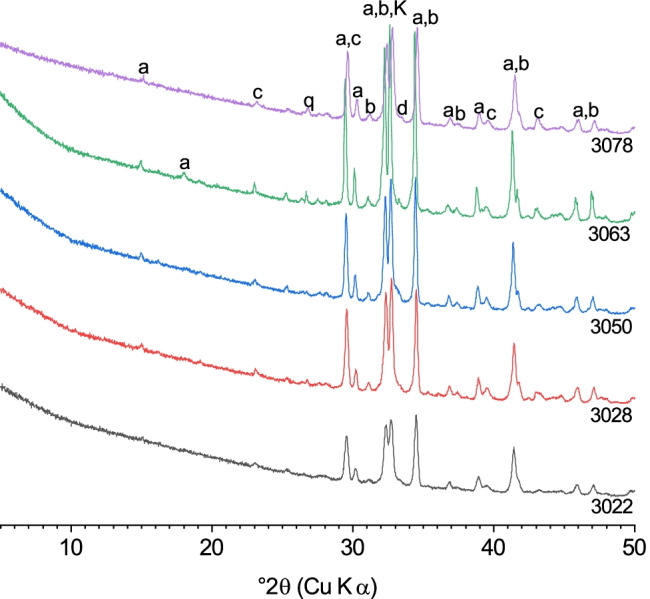
X-ray diffractograms of the cement samples 3022, 3028, 3050, 3063, and 3078 after KOSH treatment: (a) alite, (b) belite, (d) aluminate, (c) calcite, (q) quartz, (K) KOH

The KOSH treatment resulted in the selective dissolution of the cements, as shown in Fig. 2. The clinker phases alite and belite remained unaffected by the KOSH treatment, while aluminate largely dissolved, and ferrite completely disappeared. In addition, the sulfates were removed from the cements. Reflections of quartz and calcite were detected in the diffractograms of the residues, meaning that quartz had remained undissolved and that calcite either had remained undissolved or had reprecipitated during evaporation of the solution after digestion, likely due to exposure to CO_2_ in the air. The relative intensity of the peak at 32.68° 2*θ* was increased compared to the untreated cements. This is the position of the main peak of potassium hydroxide (KOH; PDF # 01–089-7389); thus, the relative increase is possibly related to precipitation of KOH from the KOSH solution during treatment.

#### XRD results of the residues after concentrated hydrochloric acid/nitric acid dissolution (conc. acid)

**Fig. 3:**
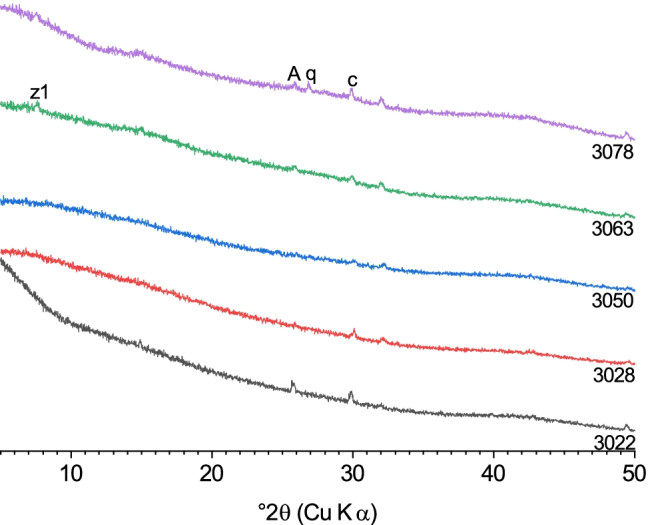
X-ray diffractograms of the cement samples 3022, 3028, 3050, 3063, and 3078 after conc. acid treatment: (A) anhydrite, (c) calcite, (q) quartz, (z1) zeolite-type phase

In contrast to the KOSH treatment, the conc. acid treatment led to a virtually complete dissolution of the initial cement phases (Fig. 3). No alite-, belite-, aluminate-, and ferrite-related peaks were detected in the diffractograms. Most of the samples were fully X-ray amorphous. The diffractogram of cement 3063 after the treatment exhibited a reflection around 7.20° 2*θ*, which could be matched with an LTA-type zeolite (PDF # 01–089-8015). Clinker 3050b had an additional peak at 9.64° 2*θ* (see Fig. S4, ESM), which can be tentatively assigned to dealuminated chabazite (PDF # 00–052-0784). The presence of these newly formed silicates in the residues is an additional indication that the clinker silicates, i.e. alite and belite, had dissolved during the conc. acid treatment. In some cases, signals with very low intensity were present around 25.4 and 29.6° 2*θ*, which were possibly caused by minor amounts of remaining anhydrite and calcite, respectively. Again, this would mean that these phases had either partly remained undissolved or have reprecipitated during evaporation after digestion of the samples.

#### XRD results of the residues after dilute nitric acid dissolution (dil. acid)

**Fig. 4:**
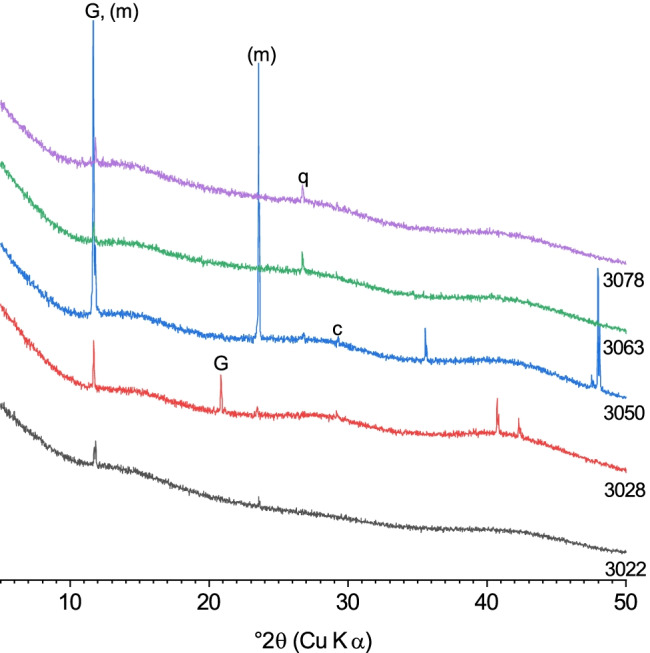
X-ray diffractograms of the cement samples 3022, 3028, 3050, 3063 and 3078 after dil. acid treatment: (G) gypsum, (m) monocarboaluminate, (c) calcite, (q) quartz

In general, the results for the cements treated with dil. acid are comparable to those of the samples treated with the conc. acid solution. Namely, the diffractograms indicate that the initial cement phases had dissolved, and the samples were virtually fully amorphous, though some of them exhibited few distinct peaks of crystalline phases (Fig. 4). Cements 3022, 3028, 3050, 3063, and 3078 contained minor amounts of gypsum, and samples 3028, 3063, and 3050 additionally contained quartz, which was present in the original cements, besides amorphous phase.

In samples 3022 and 3050, additional peaks were present at 11.8° 2*θ* and 23.59° 2*θ*, which are assigned to tetracalcium monocarboaluminate (Ca_4_Al_2_(OH)_12_[CO_3_]·5H_2_O; PDF # 01–087-0493). The carbonate ion in monocarboaluminate is readily replaced by nitrate [16]; thus, its NO_3_-exchanged form (“NO_3_-AFm”) could have been expected after treatment with nitric acid. The present results, however, indicate that heating at 130 °C for 48 h had effectively removed nitrate from the samples, so that monocarboaluminate formed, with the required CO_2_ likely provided by the atmosphere.

### Results of ^87^Sr/^86^Sr isotope analyses

#### Concentrated hydrochloric acid/nitric acid dissolution (conc. acid) and sieving of the cements

**Fig. 5:**
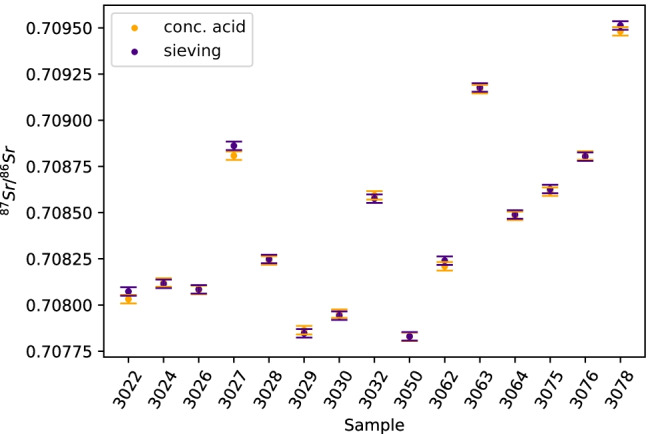
^87^Sr/^86^Sr isotope ratios of fifteen cement samples after conc. acid treatement (orange) and after the sieving method (purple); error bars represent expanded uncertainty (*U*, *k* = 2).

In Fig. 5, the ^87^Sr/^86^Sr isotope ratios of the cements digested by conc. acid treatment are compared with those of the same cements after sieving and subsequent digestion in conc. acid. The isotope ratios obtained with these two methods excellently agree within the stated uncertainties for almost all cements, in line with the XRD analyses, which showed that sieving did not remove the calcium sulfate additions from the cements, nor significantly changed their phase assemblages in other ways.

#### KOSH treatement

**Fig. 6:**
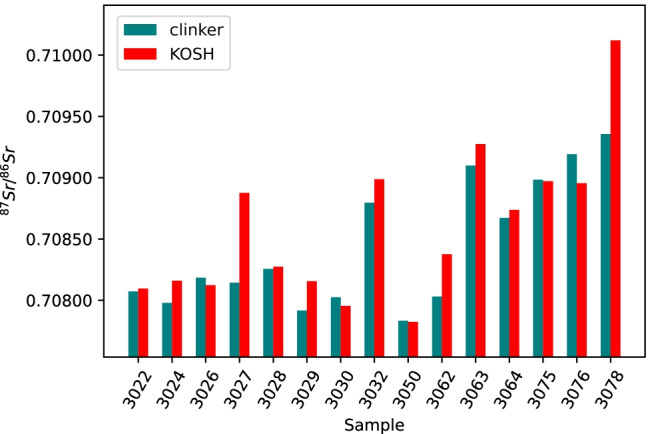
Comparison of ^87^Sr/^86^Sr isotope ratios from fifteen cement samples after KOSH treatment (red) and fifteen corresponding clinker samples after conc. acid treatment (blue-green)

Sr isotope ratios for cement samples after KOSH method and subsequent conc. acid digestion were compared to the corresponding clinkers after conc. acid treatement, as shown in Fig. 6. For samples 3027, 3029, 3062, and 3078, the KOSH treatment gave considerably different ratios for the cements and the clinkers, the ^87^Sr/^86^Sr isotope ratios of the cements being much higher than those obtained for the corresponding clinker samples. In some cases, such as 3026, 3030, and 3076, lower ratios were obtained for the cements than for the corresponding clinker samples. Therefore, there is no general trend discernable in the data. However, a paired *t*-test applied to the data shows that the difference between KOSH-treated cements and the corresponding clinker samples is statistically significant (*t*(14) =  − 2.15, *p* = 0.05). The results of all calculated *t*-tests can be found in Tables S4–S7 in the ESM.

#### Concentrated hydrochloric acid/nitric acid dissolution (conc. acid)

**Fig. 7:**
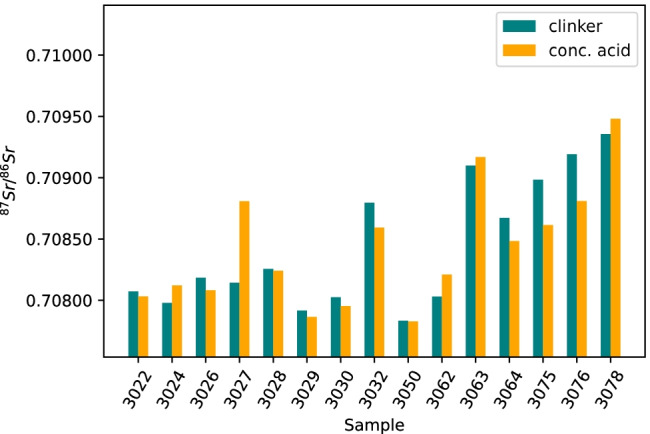
Comparison of ^87^Sr/^86^Sr isotope ratios from fifteen cement samples after conc. acid treatment (orange) and fifteen corresponding clinker samples after conc. acid treatment (blue-green)

When comparing the ^87^Sr/^86^Sr isotope ratios of the cement samples after conc. acid treatment with the corresponding clinker samples, the situation is different (Fig. 7). The conc. acid treatment gave a considerably higher ratio for only one cement sample (3027); slightly higher ratios for the four cement samples 3024, 3062, 3063, and 3078; and a lower ratio for the other samples, especially for 3075 and 3076. The results of the *t*-test showed no statistically significant difference between the cement samples after the conc. acid treatment and the clinker samples (*t*(14) = 0.26, *p* = 0.80).

A comparison was also performed between the cements after sieving and subsequent conc. acid treatment and the corresponding clinker samples after conc. acid digestion. The results are shown in Fig. S5 in the ESM. Both sieving and conc. acid treatment of the cements yielded very similar Sr isotope data (cf. Figure 5). This confirms the results of the XRD measurements.

#### Dilute nitric acid dissolution (dil. acid)

**Fig. 8:**
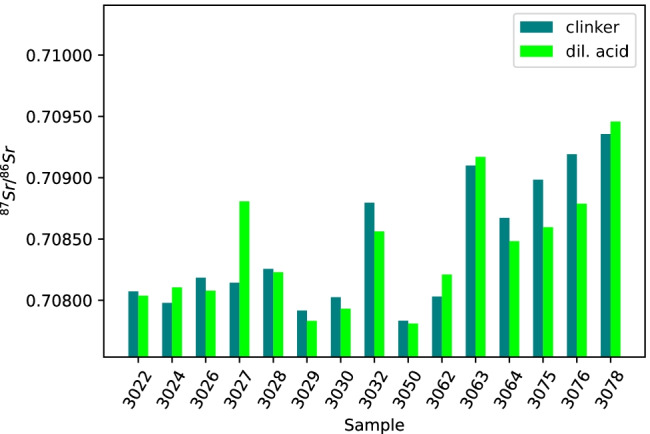
Comparison of ^87^Sr/^86^Sr isotope ratios from fifteen cement samples after dil. acid treatment (fluorescent green) and fifteen corresponding clinker samples after conc. acid treatment (blue-green)

In Fig. 8, the ^87^Sr/^86^Sr isotope ratios of the cements digested by dil. acid treatment are compared with those of the corresponding clinkers after digestion in conc. acid. The cements after dil. acid treatment show the same trend as the cements after conc. acid treatment. In fact, the dil. acid treatment gave a much higher ratio for only one cement (3027), slightly higher for the four cements 3024, 3062, 3063, and 3078, and lower ratio for the other samples, especially for 3075 and 3076. There was no significant difference between the samples after dil. acid treatment and the clinker samples (*t*(14) = 0.45, *p* = 0.66).

#### Discussion

The selection of the most suitable sample preparation procedure was realised by comparing the ^87^Sr/^86^Sr isotope ratios of differently treated cement samples (processed cements) with the corresponding clinker samples (see Tables S1 and S2 in the ESM). The final goal was to find a procedure for preparing cement in such a way that the ^87^Sr/^86^Sr isotope ratio gives the closest value to the ^87^Sr/^86^Sr isotope ratio of the corresponding clinker, but also to fulfil the requirements regarding efficiency, e.g. time consumption.

To better compare the four different procedures applied to fifteen pairs of samples, we reduced the data for each sample. The ^87^Sr/^86^Sr isotope ratio of each processed cement was used to compute the absolute difference to the corresponding clinker sample according to

1 $$\Delta_{\mathrm{abs}}=\left|{\left(\frac{{}^{87}\mathrm{Sr}}{{}^{86}\mathrm{Sr}}\right)}_{\mathrm{cement}}-{\left(\frac{{}^{87}\mathrm{Sr}}{{}^{86}\mathrm{Sr}}\right)}_{\mathrm{clinker}}\right|$$

**Fig. 9:**
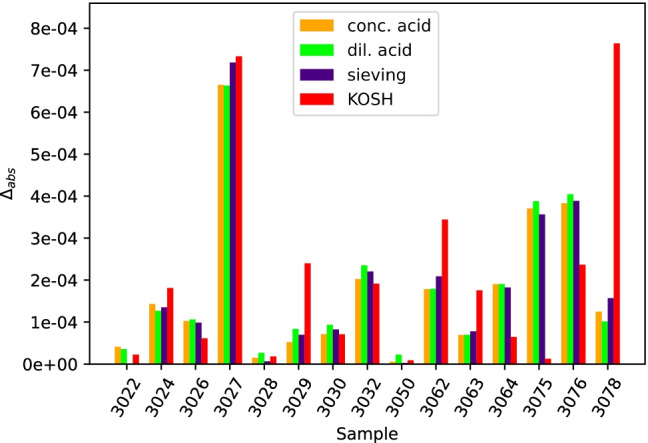
Comparison of all four methods on fifteen processed cement samples presented as the absolute difference between the ^87^Sr/^86^Sr isotope ratio of the processed cement and the ^87^Sr/^86^Sr isotope ratio of the corresponding clinker

The so-computed differences (see Table S3 in the ESM) are shown in Fig. 9 for all four methods applied to fifteen processed cement samples.

**Fig. 10:**
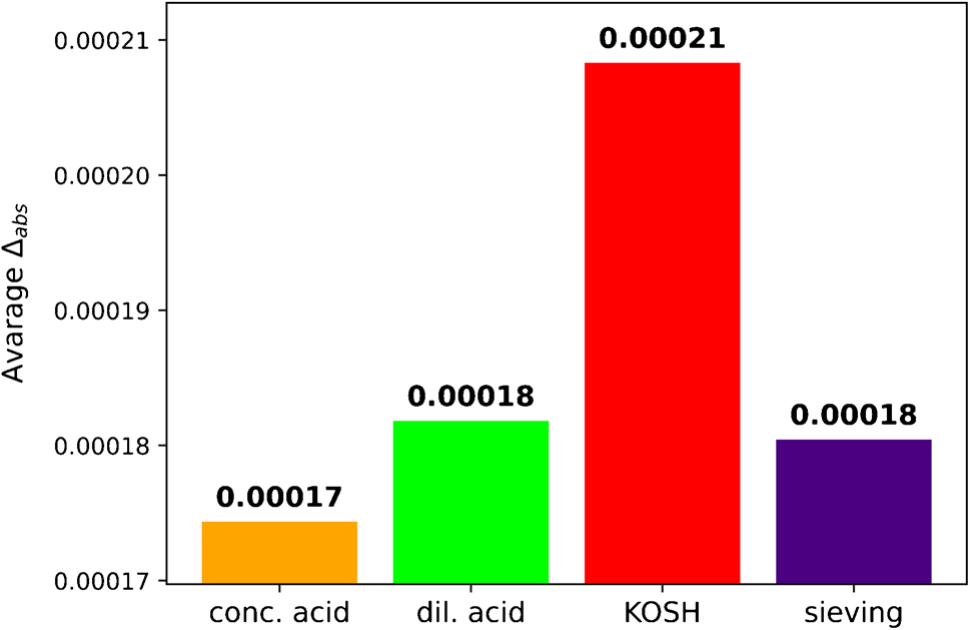
Comparison of all methods: average absolute difference of the ^87^Sr/^86^Sr isotope ratios of the processed cements and the corresponding clinkers

For further data reduction, the average of *Δ*_abs_ was calculated for each sample treatment. The results are shown in Fig. 10. The lower the average absolute difference is, the closer are the ^87^Sr/^86^Sr isotope ratios determined for the cements to those of the corresponding clinkers on average, i.e. the better the method met the requirement to reflect the ^87^Sr/^86^Sr isotope ratio of the clinker. The average absolute differences were 0.00021, 0.00018, 0.00018, and 0.00017, for KOSH, sieving, dil. acid, and conc. acid respectively.

A possible explanation for the comparatively large average ${\Delta }_{\mathrm{abs}}$ resulting from the KOSH treatment is contamination during the treatment. The measured procedural Sr blank for the KOSH method was 75 ng/g, resulting in 3.4 µg of Sr in a standard batch of KOSH solution (45 g of KOSH solution). Assuming that all Sr in the KOSH solution is transferred to the cement, the result is 3.4 µg of Sr contamination per 1 g of treated cement. This amount can have a significant impact on the measured total ^87^Sr/^86^Sr isotope ratios, because the Sr contents of the cements were in the range of 100–1200 µg/g, leading to a total Sr mass of 100 to 1200 µg per sample treatment. However, an influence of the ^87^Sr/^86^Sr isotope ratio of the KOSH solution/ blank does not fully explain the observed deviations, because if it had been the sole influence, the contamination from the blank would shift the samples isotope ratios towards the blank isotope ratio. However, inspection of Fig. 6 does not reveal a systematic trend, neither for a low, high, or seawater isotopic composition of the blank. Thus, evidently other factors play a role here, which, however, could not be elucidated in the present study.

To rank the usefulness of the four methods, we used five parameters (Table 1). The first parameter is the average absolute difference (average *Δ*_abs_) between the ^87^Sr/^86^Sr isotope ratios of the processed cements and the corresponding clinkers. This parameter was introduced because it reflects extreme differences between the sample pairs (cement and corresponding clinker). Conc. acid treatment exhibited the lowest average *Δ*_abs_, meaning this method yielded the value closest to the target on average. The KOSH treatment showed the highest average *Δ*_abs_. For dil. acid treatment and sieving, the average *Δ*_abs_ was approximately the same, intermediate between the values of conc. acid treatment and KOSH treatment.

**Table 1:** Comparison of all methods regarding the difficulty of preparation, time consumption in hours and number of prepared samples per workday

Method	Average difference from clinker	*N*_1_ samples below threshold	Difficulty of preparation	Time consumption in hours	*N*_2_ samples per workday
conc. acid	0.00017	5	Low	2	15
dil. acid	0.00018	4	Low	2	15
Sieving 11 µm	0.00018	4	Medium	8*	3**
KOSH	0.00021	6	High	6	5

*Including cleaning the sieves; **limited by the number of available sieves.

The second parameter was the number (*N*_1_) of samples (cement-clinker pairs) which gave a *Δ*_abs_ below a specified threshold. Three times the expanded uncertainty (3 × *U*) was selected as the threshold. Every sample pair (cement and clinker) was evaluated within every sample preparation procedure. The higher the number *N*_1_ of samples that fit the criterion, the better the preparation procedure. The square sum approach was used to assess the combined standard uncertainty, *u*_c_, of the ^87^Sr/^86^Sr isotope ratio results. The following uncertainty components were identified: repeatability of a single ^87^Sr/^86^Sr measurement in a sample, repeatability of ^87^Sr/^86^Sr measurement in measured NIST SRM 987, the bias to the reference value for ^87^Sr/^86^Sr measurement in NIST SRM 987 (value published in GeoReM database [14]), the experimental reproducibility of independently processed samples, the bias of the measured ^87^Sr/^86^Sr ratio in a processed AGV-2a sample versus the reference value (value published in GeoReM database [14]), and the bias of measured ^87^Sr/^86^Sr ratio in processed NASS-6 sample versus literature value. The expanded uncertainty (*U* = *k *∙ *u*_c_, with *k* = 2) of the method for obtaining conventional ^87^Sr/^86^Sr isotope ratios was calculated to be 0.000023. Based on these calculations, *N*_1_ was 6, 5, 4, and 4 for KOSH, conc. acid, sieving, and dil. acid treatment, respectively.

The other three parameters were evaluated by the analysts and include the complexity of preparation, the time consumption in hours, and the number *N*_2_ of prepared samples per workday. Thus, these parameters are strictly applicable only to the conditions of the present study, but they can serve as an estimate for other laboratories too. Conc. acid and dil. acid treatment were neither difficult nor time consuming, and *N*_2_ was 15. Sieving and KOSH treatment were more difficult to perform, particularly the KOSH treatment, due to the involved compounds, and the need for repeating several steps, e.g. dissolving, centrifugation, and drying. Furthermore, for KOSH treatment and sieving, the time consumption were higher, and the number of samples which can be handled per workday were lower. This especially applied for sieving, since cleaning of the sieves after use was time consuming, and the number of available sieves was a limiting factor.

## Summary and conclusions

The present study shows that with the employed setup and conditions for the sieving method, satisfying results could not be achieved. Sieving was not suitable to separate coarser clinker particles from the finer calcium sulfate particles of cement, and therefore, the additives could not be removed before Sr isotope analysis. This was confirmed by Sr isotope analysis on MC-TIMS, as well as by XRD analysis, which showed the calcium sulfates to be present in the coarse fraction after sieving. Future work should investigate whether air-jet sieving is more appropriate for the present purpose.

XRD results confirmed that the KOSH method successfully led to selective dissolution of clinker phases and cement components, with the aluminate almost completely dissolved, and the calcium sulfates and ferrite completely removed. The clinker phases alite and belite remained largely unaffected by the KOSH treatment. Thus, only KOSH has proven effective out of the two employed approaches to remove the calcium sulfate additives from the cements.

XRD showed that the conc. acid method led to a virtually complete breakdown of the initial phases, where all alite-, belite-, aluminate-, and ferrite-related peaks disappeared. That means that all Sr-bearing compounds in the cement were dissolved except for insoluble minor constitutents (e.g. quartz). The dil. acid method yielded very similar results.

Despite the complete removal of the calcium sulfates from the cements, due to reasons that could not be clarified in the present study, the average deviation between the ^87^Sr/^86^Sr isotope ratios of the cements treated with the KOSH solution and the corresponding clinkers (*Δ*_abs_) was the largest in the present study, although the KOSH treatment also returns the most pairs with a *Δ*_abs_ below the chosen threshold (3 × *U*). This is somehow contradictory and cannot be explained by blank issues only. The partial dissolution and reprecipitation during KOSH treatment might dissolve varying Sr reservoirs depending on the individual cement sample and thus leading to this behaviour.

The fact that the average *Δ*_abs_ was lowest for the conc. acid treatment and that the *t*-test showed that the difference between the ^87^Sr/^86^Sr isotope ratios of the cement samples treated with conc. acid and the corresponding clinkers was not statistically significant, while it was for cements after KOSH treatment, strongly argues for the sample preparation with conc. acid. Treatment with dil. acid led to slightly less satisfactory results, i.e. a slightly higher average *Δ*_abs_ and a lower number of samples with a ^87^Sr/^86^Sr ratios difference between cement and clinker below the threshold.

It is thus concluded that dissolution in conc. acid (concentrated hydrochloric acid/nitric acid) yields satisfactory results and is currently the most appropriate sample preparation method for determination the ^87^Sr/^86^Sr isotope ratios of Portland cements (CEM I), compared to the other methods employed in the present study. The obtained values can serve as a proxy for the ^87^Sr/^86^Sr isotope ratio of the clinker in the cement and can thus be used for cement provenance studies.

## Supplementary Information

Below is the link to the electronic supplementary material.

Supplementary file1 (DOCX 640 KB)

## Data Availability

The data used to support the findings of this study is available within the article and the associated Supplementary Information file. The data is also available from the corresponding author upon reasonable request.

## Abbreviations

CEM I: Ordinary Portland cement as specified in EN 197–1
conc. acid: Concentrated HNO_3_ and HCl 1:1 *v/v*
dil. acid: 1 mol L^−1^ HNO_3_
FDG: Flue gas desulfurisation
ICP-MS: Inductively coupled plasma mass spectrometry
KOSH: Selective dissolution of clinker phases using KOH/sucrose solution
MC-TIMS: Multi collector thermal ionisation mass spectrometry
Sieving: Sieving on 11-μm sieve
XRD: X-ray diffraction

## References

[1] Elhacham E, Ben-Uri L, Grozovski J, Bar-On YM, Milo R (2020). Global human-made mass exceeds all living biomass. Nature.

[2] Taylor HFW (2009). Cement chemistry.

[3] Kazlagic A, Vogl J, Gluth GJG, Stephan D (2021). Provenancing of cement using elemental analyses and isotope techniques – the state-of-the-art and future perspectives. J Anal At Spectrom.

[4] Goguel RL, Stjohn DA (1993). Chemical-identification of Portland cements in New-Zealand concretes .1. Characteristic differences among New-Zealand cements in minor and trace-element chemistry. Cem Concr Res..

[5] Potgieter JH, Potgieter SS, McCrindle RI, Tamas FD (2003). Fingerprinting of South African cement clinkers and gypsum as a tool for cement identification purposes. Adv Cem Res.

[6] Potgieter-Vermaak SS, Potgieter JH, Worobiec K, van Grieken R, Majanovic L, Moeketsi S (2007). Fingerprinting of South African ordinary Portland cements, cement blends and mortars for identification purposes - discrimination with starplots and PCA. Cem Concr Res.

[7] Kasamatsu M, Igawa T, Suzuki S, Suzuki Y (2018). Forensic discrimination of concrete pieces by elemental analysis of acid-soluble component with inductively coupled plasma-Mass Spectrometry. Anal Sci.

[8] Pirrie D, Pidduck AJ, Crean DE, Nicholls TM, Awbery RP (2019). Identification and analysis of man-made geological product particles to aid forensic investigation of provenance in the built environment. Forensic Sci Int.

[9] Telschow S, Frandsen F, Theisen K, Dam-Johansen K (2012). Cement formation-a success Story in a black box: high temperature phase formation of Portland cement clinker. Ind Eng Chem Res.

[10] Locher FW. Cement : principles of production and use. Düsseldorf: Bau+Technik Gmbh; 2006. p. 535.

[11] Graham IJ, Goguel RL, St John DA (2000). Use of strontium isotopes to determine the origin of cement in concretes - case examples from New Zealand. Cem Concr Res.

[12] Gutteridge WA (1979). Dissolution of the interstitial phases in Portland-cement. Cem Concr Res.

[13] Faure G, Mensing TM. Isotopes : principles and applications. 3rd ed. Hoboken: John Wiley & Sons, Inc.; 2013.

[14] Jochum KP, Nohl U, Herwig K, Lammel E, Stoll B, Hofmann AW (2005). GeoReM: a new geochemical database for reference materials and isotopic standards. Geostand Geoanal Res.

[15] Gates-Rector S, Blanton T (2019). The Powder Diffraction File: a quality materials characterization database. Powder Diffr.

[16] Balonis M, Medala M, Glasser FP (2011). Influence of calcium nitrate and nitrite on the constitution of AFm and AFt cement hydrates. Adv Cem Res.

