# Supplementary material for: Development of a sample preparation procedure for Sr isotope analysis of Portland cements
Source: Anal Bioanal Chem. 2022 Jan 14;414(15):4379–89. doi: 10.1007/s00216-021-03821-7 (PMC9142479; doi:10.1007/s00216-021-03821-7)
Supplement: Supplementary file 1 — Supplementary file1 (DOCX 640 KB) [file 216_2021_3821_MOESM1_ESM.docx]

***SUPPLEMENTARY INFORMATION FOR*:**

***Development of a sample preparation procedure for Sr isotope analysis of Portland cements***

Anera Kazlagić^1^, Francesco F. Russo^1^, Jochen Vogl^1^, Patrick Sturm^2^, Dietmar Stephan^3^,
Gregor J. G. Gluth^2^

^1^ Federal Institute for Materials Research and Testing, Division 1.1 Inorganic Trace Analysis, Richard-Willstäter-Straße 11, 12489 Berlin, Germany

^2^ Federal Institute for Materials Research and Testing, Division 7.4 Technology of Construction Materials, Unter den Eichen 87, 12205 Berlin, Germany

^3^ Technische Universität Berlin, Department of Civil Engineering, Building Materials and Construction Chemistry, Gustav-Meyer-Allee 25, 13355 Berlin, Germany

Correspondence to: anera.kazlagic@bam.de

**Contents**

AbbreviationsS-2

EquationS-2

Fig. S1S-3

Fig. S2S-4

Fig. S3S-5

Fig. S4S-6

Fig. S5S-7

Table S1S-8

Table S2S-9

Table S3S-10

Table S4S-11

Table S5S-12

Table S6S-13

Table S7S-14

Abbreviations:

Sieving – sieving on 11-µm sieve

KOSH - potassium hydroxide/sucrose solution

Conc. acid - Concentrated hydrochloric acid/nitric acid dissolution

Dil. acid - Dilute nitric acid dissolution

Equation:

$$\begin{aligned} \Delta_{abs}=\left| \left( \frac{{}^{87}{Sr}}{{}^{86}{Sr}} \right)_{cement}-\left( \frac{{}^{87}{Sr}}{{}^{86}{Sr}} \right)_{clinker} \right|\#\left( 1 \right) \end{aligned}$$

Legend:

Δ_abs -_ Absolute difference of processed cement from the clinker

(^87^Sr/^86^Sr)_cement_ – Sr isotope ratio of processed cement

(^87^Sr/^86^Sr)_clinker_ – Sr isotope ratio of the clinker

**Fig. S1.** X-ray diffractogram of non-treated cement (directly on top of the sample code) and sieved cement (on top of the respective non-treated pattern) for samples 3022, 3024, 3026, 3027 and 3028. G = Gypsum, A = Anhydrite.

**Fig. S2.** X-ray diffractogram of non-treated cement (directly on top of the sample code) and sieved cement (on top of the respective non-treated pattern) for samples 3029, 3030,3032, 3050 and 3062. G = Gypsum, A = Anhydrite.

**Fig. S3.** X- ray diffractogram of non-treated cement (directly on top of the sample code) and sieved cement (on top of the respective non-treated pattern) for samples 3063, 3064, 3075, and 3078. G = Gypsum, A = Anhydrite.


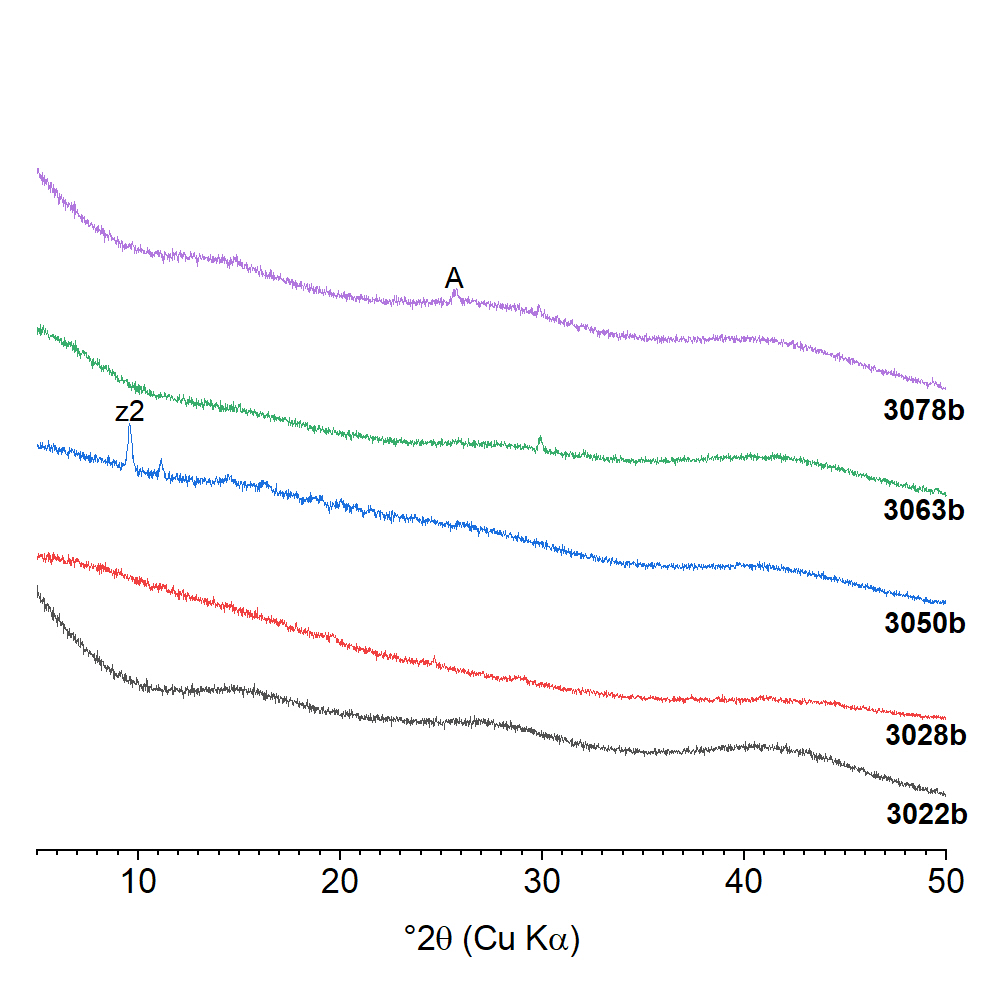


**Fig*.* S4.** X-ray diffractograms of the clinker samples 3022b, 3028b, 3050b, 3063b and 3078b after conc. acid treatment; A = anhydrite, z2 = zeolite-type phase.


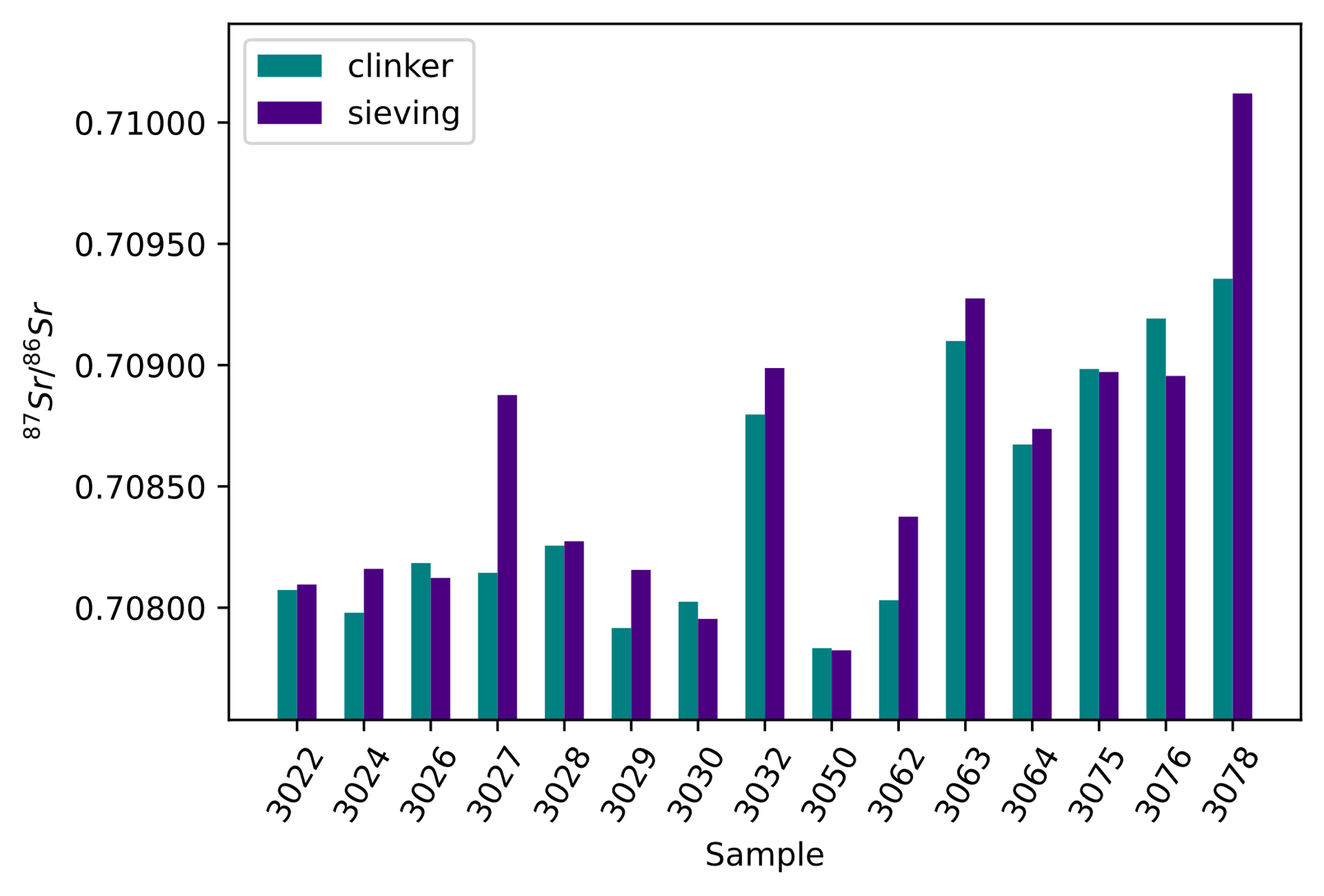


**Fig. S5.** Comparison of ^87^Sr/^86^Sr isotope ratios from fifteen cement samples prepared with sieving and subsequent conc. acid treatment (purple) and fifteen corresponding clinker samples after conc. acid treatment (blue-green).

**Table S1.** ^87^Sr/^86^Sr isotope ratio of processed clinkers, and cements after the KOSH and sieving treatment and clinker.^[[1]](#footnote-1)^ 2SE represents 2 x Standard Error (Standard Deviation divided by the square root of number of measured isotope ratios).

| Sample | ^87^Sr/^86^Sr (clinker) | 2SE x 10^‑5^ (clinker) | ^87^Sr/^86^Sr (KOSH) | 2SE x 10^‑5^ (KOSH) | ^87^Sr/^86^Sr (Sieving) | 2SE x 10^‑5^ (Sieving) |
| --- | --- | --- | --- | --- | --- | --- |
| 3022 | 0.70807 | 1 | 0.70810 | 1 | 0.70807 | 1 |
| 3024 | 0.70798 | 1 | 0.70816 | 1 | 0.70811 | 1 |
| 3026 | 0.70818 | 3 | 0.70812 | 7 | 0.70809 | 1 |
| 3027 | 0.70814 | 1 | 0.70888 | 6 | 0.70886 | 1 |
| 3028 | 0.70826 | 1 | 0.70827 | 1 | 0.70825 | 2 |
| 3029 | 0.70792 | 1 | 0.70816 | 1 | 0.70785 | 1 |
| 3030 | 0.70802 | 1 | 0.70795 | 1 | 0.70794 | 1 |
| 3032 | 0.70880 | 1 | 0.70899 | 1 | 0.70858 | 1 |
| 3050 | 0.70783 | 1 | 0.70782 | 1 | 0.70783 | 1 |
| 3062 | 0.70803 | 3 | 0.70838 | 1 | 0.70824 | 1 |
| 3063 | 0.70910 | 1 | 0.70927 | 1 | 0.70918 | 1 |
| 3064 | 0.70867 | 1 | 0.70874 | 1 | 0.70849 | 1 |
| 3075 | 0.70898 | 1 | 0.70897 | 1 | 0.70863 | 1 |
| 3076 | 0.70919 | 1 | 0.70896 | 1 | 0.70880 | 1 |
| 3078 | 0.70936 | 1 | 0.71012 | 2 | 0.70951 | 1 |

**Table S2.**  ^87^Sr/^86^Sr isotope ratio of processed cements after the dil. acid and conc. acid treatment.

| Sample | ^87^Sr/^86^Sr (Dil. Acid) | 2SE x 10^‑5^ (Dil. Acid) | ^87^Sr/^86^Sr (Conc. Acid) | 2SE x 10^‑5^ (Conc. Acid) |
| --- | --- | --- | --- | --- |
| 3022 | 0.70804 | 1 | 0.70803 | 1 |
| 3024 | 0.70811 | 1 | 0.70812 | 1 |
| 3026 | 0.70808 | 1 | 0.70808 | 1 |
| 3027 | 0.70881 | 1 | 0.70881 | 3 |
| 3028 | 0.70823 | 1 | 0.70824 | 1 |
| 3029 | 0.70783 | 1 | 0.70786 | 1 |
| 3030 | 0.70793 | 1 | 0.70795 | 2 |
| 3032 | 0.70856 | 1 | 0.70859 | 1 |
| 3050 | 0.70781 | 1 | 0.70783 | 1 |
| 3062 | 0.70821 | 1 | 0.70821 | 1 |
| 3063 | 0.70917 | 1 | 0.70917 | 3 |
| 3064 | 0.70848 | 1 | 0.70848 | 1 |
| 3075 | 0.70860 | 1 | 0.70861 | 1 |
| 3076 | 0.70879 | 1 | 0.70881 | 1 |
| 3078 | 0.70946 | 1 | 0.70948 | 1 |

**Table S3.** Absolute difference Δ_abs_ between the ^87^Sr/^86^Sr isotope ratio of the cement and the ^87^Sr/^86^Sr isotope ratio of the corresponding clinker for the four investigated preparation methods. Δ_abs_ was calculated using equation (1).

| Sample | Δ*_abs_* (KOSH) x 10^-5^ | Δ*_abs_* (sieving) x 10^-5^ | Δ*_abs_* (dil. acid) x 10^-5^ | Δ*_abs_* (conc. acid) x 10^-5^ |
| --- | --- | --- | --- | --- |
| 3022 | 2 | 0.03 | 4 | 4 |
| 3024 | 18 | 14 | 13 | 14 |
| 3026 | 6 | 10 | 11 | 10 |
| 3027 | 73 | 72 | 66 | 66 |
| 3028 | 2 | 1 | 3 | 2 |
| 3029 | 24 | 7 | 8 | 5 |
| 3030 | 7 | 8 | 9 | 7 |
| 3032 | 19 | 22 | 23 | 20 |
| 3050 | 1 | 0.3 | 2 | 1 |
| 3062 | 34 | 21 | 18 | 18 |
| 3063 | 18 | 8 | 7 | 7 |
| 3064 | 6 | 18 | 19 | 19 |
| 3075 | 1 | 36 | 39 | 37 |
| 3076 | 24 | 39 | 40 | 38 |
| 3078 | 76 | 16 | 10 | 12 |

**Table S4.** Results of the paired t-test for ^87^Sr/^86^Sr isotope ratios of clinker and KOSH ^87^Sr/^86^Sr isotope ratios of cements as calculated with Excel (Office 365).

| t-Test: Paired Two Sample for Means | | |
| --- | --- | --- |
|  | *^87^Sr/^86^Sr Clinker* | *^87^Sr/^86^Sr KOSH* |
| Mean | 0.708436 | 0.708592 |
| Variance | 2.74E-07 | 3.8E-07 |
| Observations | 15 | 15 |
| Pearson Correlation | 0.891731 | |
| Hypothesized Mean Difference | 0 | |
| df | 14 | |
| t Stat | -2.15852 | |
| P(T<=t) one-tail | 0.024369 | |
| t Critical one-tail | 1.76131 | |
| P(T<=t) two-tail | 0.048737 | |
| t Critical two-tail | 2.144787 | |

**Table S5.** Results of the paired t-test for clinker ^87^Sr/^86^Sr isotope ratios and sieving ^87^Sr/^86^Sr isotope ratios of cements as calculated with Excel (Office 365).

| t-Test: Paired Two Sample for Means | | |
| --- | --- | --- |
|  | *^87^Sr/^86^Sr Clinker* | *^87^Sr/^86^Sr Sieved* |
| Mean | 0.708436 | 0.708429 |
| Variance | 2.74E-07 | 2.48E-07 |
| Observations | 15 | 15 |
| Pearson Correlation | 0.865751 | |
| Hypothesized Mean Difference | 0 | |
| df | 14 | |
| t Stat | 0.107146 | |
| P(T<=t) one-tail | 0.458097 | |
| t Critical one-tail | 1.76131 | |
| P(T<=t) two-tail | 0.916194 | |
| t Critical two-tail | 2.144787 | |

**Table S6.** Results of the paired t-test for clinker ^87^Sr/^86^Sr isotope ratios and dil. acid ^87^Sr/^86^Sr isotope ratios of cements as calculated with Excel (Office 365).

| t-Test: Paired Two Sample for Means | | |
| --- | --- | --- |
|  | *^87^Sr/^86^Sr Clinker* | *^87^Sr/^86^Sr Dil* |
| Mean | 0.708436 | 0.708406 |
| Variance | 2.74E-07 | 2.41E-07 |
| Observations | 15 | 15 |
| Pearson Correlation | 0.873467 | |
| Hypothesized Mean Difference | 0 | |
| df | 14 | |
| t Stat | 0.447992 | |
| P(T<=t) one-tail | 0.330507 | |
| t Critical one-tail | 1.76131 | |
| P(T<=t) two-tail | 0.661014 | |
| t Critical two-tail | 2.144787 | |

**Table S7.** Results of the paired t-test for clinker ^87^Sr/^86^Sr isotope ratios and conc. acid ^87^Sr/^86^Sr isotope ratios of cements as calculated with Excel (Office 365).

| t-Test: Paired Two Sample for Means | | |
| --- | --- | --- |
|  | *^87^Sr/^86^Sr Clinker* | *^87^Sr/^86^Sr conc* |
| Mean | 0.708436 | 0.708419 |
| Variance | 2.74E-07 | 2.41E-07 |
| Observations | 15 | 15 |
| Pearson Correlation | 0.877963 | |
| Hypothesized Mean Difference | 0 | |
| df | 14 | |
| t Stat | 0.259105 | |
| P(T<=t) one-tail | 0.399662 | |
| t Critical one-tail | 1.76131 | |
| P(T<=t) two-tail | 0.799325 | |
| t Critical two-tail | 2.144787 | |

1. Each cement sample was loaded on a single filament, and the standard deviation was then calculated from minimum 100, maximum 200 measured isotope ratios. [↑](#footnote-ref-1)
